# Effect of Welding Speed on Microstructure Evolution and Mechanical Properties of Friction Stir Welded 2198 Al-Cu-Li Alloy Joints

**DOI:** 10.3390/ma15030969

**Published:** 2022-01-27

**Authors:** Wenyan Zhang, Yuqing Mao, Ping Yang, Ning Li, Liming Ke, Yu Chen

**Affiliations:** National Defence Key Discipline Laboratory of Light Alloy Processing Science and Technology, Nanchang Hangkong University, Nanchang 330063, China; zwy18270409466@163.com (W.Z.); yp950829@163.com (P.Y.); lining19961216@163.com (N.L.); chenyu@163.com (Y.C.)

**Keywords:** 2198 Al-Cu-Li alloy, friction stir welding, welding speed, microstructure evolution, mechanical properties

## Abstract

In the present study, 2198 Al-Cu-Li alloys were successfully friction stir welded by using various welding speed ranges of 90~180 mm/min with an invariable rotation speed of 950 r/min. The effect of welding speed on microstructure evolution and mechanical properties of the joints was investigated. The results show that, with the welding speed decreasing, the size of the nugget zone (NZ) first increases and then decreases due to different welding temperatures. At a welding speed of 150 mm/min, the size of the NZ in all joints is the biggest and the “S” curve disappears. The equiaxed grains are finer, attributed to a higher degree of dynamic recrystallization, and a larger number of fine reprecipitated phase (δ’, β’ phases) particles are dispersively distributed in the NZ. Correspondingly, the joints have the highest tensile properties, and the tensile strength, yield strength and elongation are, respectively, 406 MPa, 289 MPa and 7.2%. However, compared to the base material, the tensile properties of all joints are reduced because a greater amount of δ’ and β’ phases particles are dissolved in the NZ. Only the joints produced at 150 mm/min are fractured in the TMAZ with detected deep dimples and tearing ridges, and a significant necking phenomenon is observed, which indicates a complete ductile fracture mode.

## 1. Introduction

Compared with traditional aluminum (Al) alloys, the third generation aluminum–lithium (Al–Li) alloy is the most desirable metal material, which is attributing to lower density [1], higher mechanical properties [2], better corrosion resistance [3] as well as better super plasticity [4]. Li is a very light metal and the solubility of the Li element in the Al alloy is very high [5]. Due to this feature, when 1% Li element is added into Al alloy, the density of the Al–Li alloy will be reduced by about 3% [6], and its elastic modulus will be increased by nearly 6% [7]. Therefore, this generation of Al–Li alloy is thought to be one of the most ideal metal materials for aerospace applications [8].

Since many large complex Al–Li alloy structures for aerospace applications are difficult to prepare by near-net-shape forming, it is often necessary to consider the joining of small pieces by welding, as one of the important joining methods. However, the traditional welding of Al–Li alloys faces many difficulties, such as weld cracking and large deformation resulting from a large residual stress [9], pores inside the weld [10], and the burning and evaporation of lithium elements [11]. Hence, as one of the solid-state welding technologies, friction stir welding (FSW), in which the base material does not melt in the welding process [12] and the welding heat input is low, can avoid the above-mentioned metallurgical defects during the fusion welding process [13], and is deemed to be the most ideal welding method for Al–Li alloy.

At present, there are some reports on FSW of Al–Li alloy as follows. Sidhar et al. [14] studied the process of FSW 1424 Al-Mg-Li alloy. Ma et al. [15] investigated the post-heat treatment process of FSW 1460 Al–Li alloy. Liu et al. [16] analyzed microstructure evolution and its effect on mechanical properties of FSWed 2060-T8 Al–Li alloy by changing rotation speed. Similarly, Chen et al. [17] discussed microstructure characterization of the FSW joint of 2099 Al–Li alloy. In addition, Zhang et al. [18] analyzed the effect of welding parameters on the formation quality of the FSW joint of 2195 Al–Li alloy, and found that the strength of the FSW joint first increased and then decreased with increasing welding speed. Mao et al. [19] found that rotation and welding speeds had an obvious impact on the microstructure evolution and metallurgical properties of FSWed 2060 Al–Li alloy joints. However, very little attention has been given to the in-depth studies on the FSW of 2198 Al–Li alloy. For example, Xing et al. [20] investigated the effect of welding speed on the mechanical properties of FSW dissimilar to 2198 and C24S Al–Li alloys joints, but their study lacked a specific analysis of dynamic recrystallization behavior and strengthening phase distribution in the joints.

Therefore, the influence of welding speed on the microstructure evolution and mechanical properties of FSW 2198 Al–Li alloy joints is presented in this study. In detail, microstructure evolution, grain size, dynamic recrystallization and the secondary precipitated phase distribution of FSW joints are discussed, and the change of mechanical properties is evaluated.

## 2. Materials and Method

**Figure 1:**
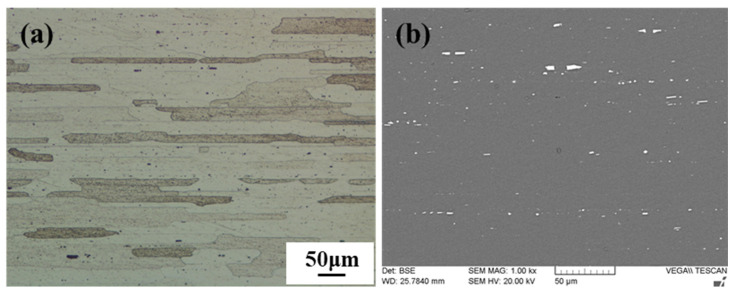
Microstructure of base material (2198 Al–Li alloy): (**a**) Microstructure; (**b**) precipitated phase distribution.

A 2 mm thick 2198-T8 Al–Li alloy was selected as the base material in the paper, and its chemical composition is listed in Table 1. Figure 1 shows the microstructure of the Al–Li alloy substrate, mainly composed of slender slate-like structures with coarse T1, θ’, δ’ and β’ particles. The Al–Li alloy sheets, processed into 200 mm × 100 mm coupons and cleaned with alcohol and acetone, were manufactured by fiction stir butt welding with welding speeds of 90, 120, 150 and 180 mm/min and an invariable rotation speed of 950 r/min. All experiments were conducted on an X53K type FSW machine, and the welding process was prepared along the rolled direction (RD) of an Al–Li alloy sheet with a 0.2 mm plunge depth and a 2° tilt angle. The FSW tool was made with H13 die steel in heat-treated conditions, and the concave shoulder diameter was 12 mm and the cylindrical pin length with 1 mm fluted pitch was 1.7 mm. The diameter of the pin root and pin top are, respectively, 4 mm and 3 mm.

The middle of the welded joints was cut into metallographic and tensile samples by a wire-cut electrical discharge machine. The grain size and recrystallization behavior of the nugget zone (NZ) in the weld, after grinding and polishing, were observed by Tescan VEGA II-LMH scanning electron microscopy (SEM) with an electron backscattered diffraction (EBSD) system. Another part of the sample was thinned into 50 μm first by mechanical polishing, and was then twin-jet electro-polished with a mixture of 25% nitric acid and 75% methanol, and the distribution of secondary strengthening phase particles in the NZ was further analyzed by Tecnai F 30G2 transmission electron microscopy (TEM). In addition, the microhardness of the joint cross section was measured by an HX-1000 Vickers hardness tester, and the test position was on the horizontal line of the center of the plate thickness with the test point interval of 0.5mm, the loading load of 0.98 N, and the loading time of 10s. The tensile properties of the joints were tested by a WDS-100 microcomputer-controlled electronic universal testing machine at room temperature with a loading speed of 2 mm/min, and then the fractured morphologies were observed by SEM technology in order to study the fracture mechanism of FSW joints.

## 3. Results and Discussion

### 3.1. Macrostructure

**Figure 2:**
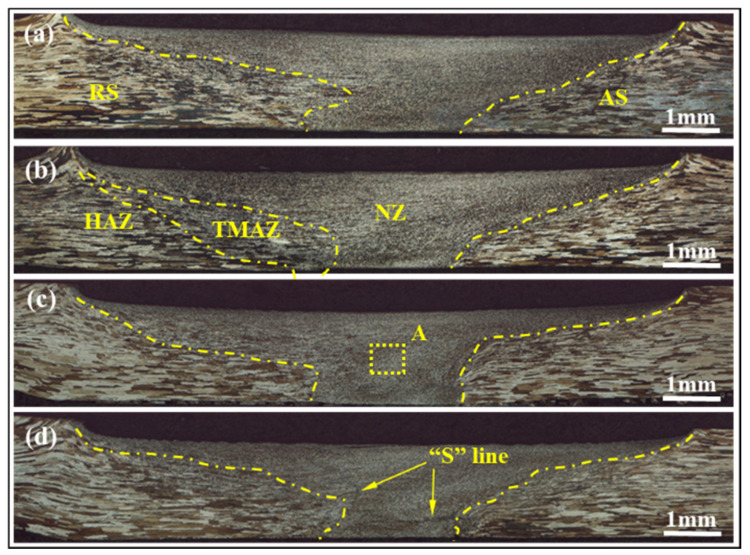
Cross-sections of FSW joints obtained at various welding speeds: (**a**) 90 mm/min; (**b**) 120 mm/min; (**c**) 150 mm/min; (**d**) 180 mm/min.

Figure 2 shows the macrostructure of FSWed joints obtained using welding speeds of 90 mm/min, 120 mm/min and 150 mm/min, 180 mm/min with an unaltered rotation speed of 950 r/min. It is observed that no grooves, holes or other welding defects exist. The well-formed weld consists of the NZ, the thermo-mechanically affected zone (TMAZ) and the heat affected zone (HAZ). As shown in Figure 2d, an “S” shaped curve appears in the weld center processed at 180 mm/min. Figure 2a,d shows the size of the NZ increases first and then decreases with decreasing welding speed. When the welding speed decreases to 150 mm/min, and the size is the largest, the internal “S” curve disappears in Figure 2c.

Moreover, the welding temperature during the FSW process may be calculated by the following formula [21]:

(1) $$\frac{T}{T_{m}}=K\left(\frac{\omega^{2}}{v\times 10^{4}}\right),$$

where T and $T_{m}$ are welding temperature and metal melting temperature, respectively; ω and V are, respectively, rotation and welding speeds; K is the correlation coefficient. It indicates that welding temperature mainly depends on the welding speed when the rotation speed is constant. Besides, the welding temperature will increase with decreasing welding speed within limits. Therefore, the welding temperature is low when a high welding speed of 180 mm/min is applied, and the weld metal is not completely plasticized, resulting in an “S” shaped curve inside the NZ in Figure 2d. When the welding speed reduces to 150 mm/min, the welding temperature can significantly increase, and sufficient plasticization degree of the weld metal results in the disappearance of the “S” curve. Another noteworthy finding is that the NZ enlarges apparently, and more plasticized metal migrates to the NZ of the weld and the metal around the extrusion zone migrates horizontally, as shown in Figure 2c. However, with continuously decreasing welding speed to 120 mm/min, the state of the contacting interface between rotating tool and surrounding metal may immediately change from adhesive friction into sliding friction [22,23]. Accordingly, the force of weld metal driving by the tool decreases, and the amount of plastic metal migrating to the NZ obviously decreases in unit time, resulting in the reduction of the size of the NZ in Figure 2a,b.

### 3.2. Microstructure

**Figure 3:**
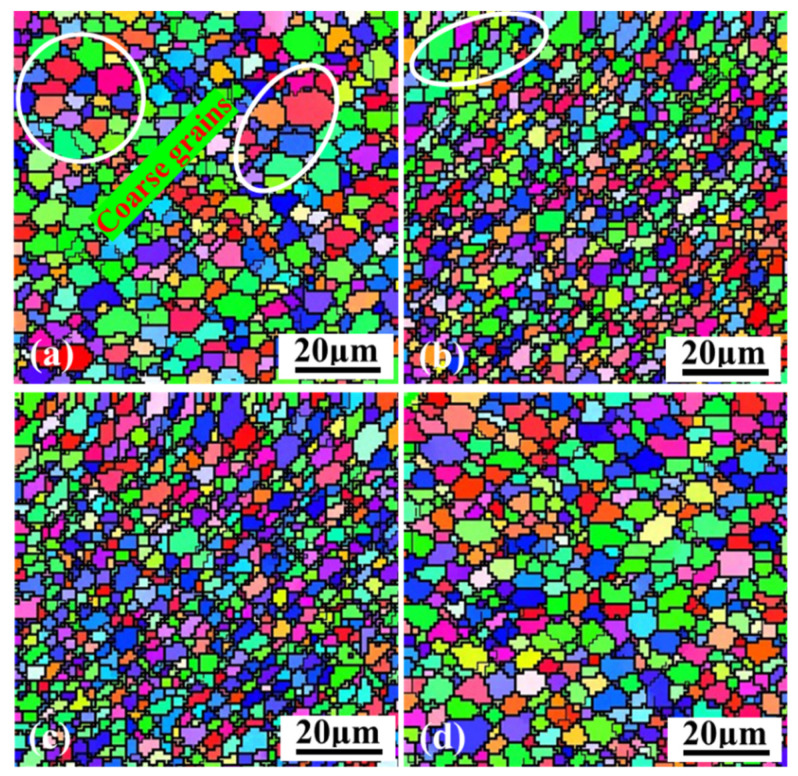
Grain size in the NZ: (**a**) 90 mm/min; (**b**) 120 mm/min; (**c**) 150 mm/min; (**d**) 180 mm/min.

Figure 3 shows grain distribution in the NZ center of the weld fabricated by various welding speeds, which is observed in section A of Figure 2c. It is seen from Figure 3 that the microstructure in different NZs is composed of fine equiaxed grains, resulting from the obvious recrystallization behavior during the FSW process [12]. However, it is also found that the size of these equiaxed grains is visibly different. The average size reduces with the welding speed decreasing from 180 to 150 mm/min, which is about 11.8 μm and 3.2 μm, respectively. What is more, with decreasing the welding speed to 120 mm/min, some fine equiaxed grains begin to grow up in Figure 3b, and the average size increases to about 4.5 μm according to the statistical result. When the lowest welding speed of 90 mm/min is applied, the grains are significantly coarsened as shown in Figure 3a.

According to Formula 1, the welding temperature in the FSW process will increase with properly decreasing welding speed, and the plastic degree of the weld metal increases. The dynamic recrystallization of the weld metal in the NZ is more sufficient [24]. As a result, the refinement degree becomes higher, and the equiaxed grains are smaller. However, when the welding speed is further lowered, the fine grains distributed in the NZ grow abnormally owing to extremely high welding temperature.

**Figure 4:**
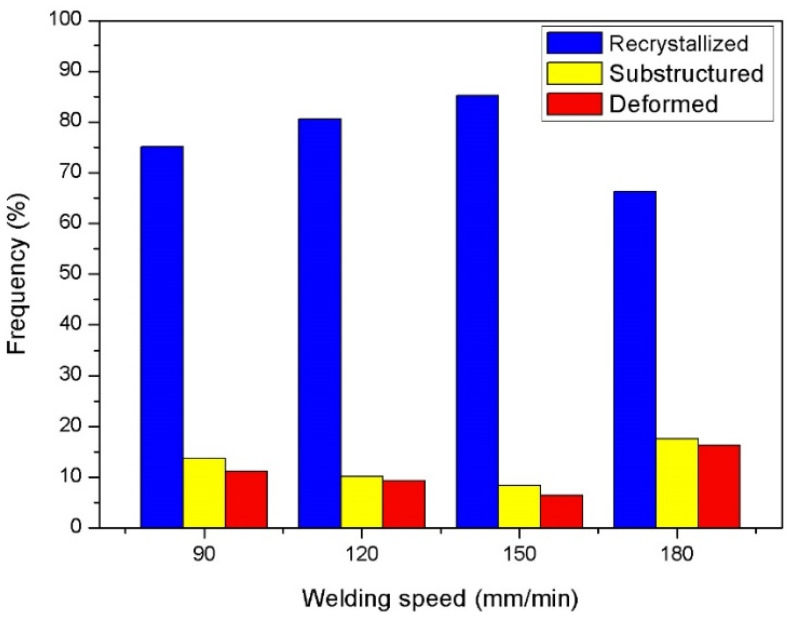
Fraction of dynamic recrystallization in the NZ.

To analyze the influence of welding speed on the dynamic recrystallization behavior of plastic metal in the NZ. The IPF maps based on the EBSD technology are counted, and the dynamic recrystallization distribution made by different welding speeds is presented in Figure 4. The blue bars represent the volume fraction of the dynamic recrystallization, and the red bars express the volume fraction of the deformation in the NZ. Seen in Figure 4, the volume fraction of the dynamic recrystallization first increases and then decreases with the welding speed decreasing from 180 mm/min to 90 mm/min. When a 180mm/min welding speed is used, the dynamic recrystallization degree is the lowest, and the volume fraction is only 66.3%. Nevertheless, it is the highest, and reaches to 85.2% at 150 mm/min. Continuously reducing the welding speed to 120 mm/min and 90 mm/min, the volume fraction decreases to 80.6% and 75.1%, respectively. It is proved that the degree of the dynamic recrystallization can be increased by decreasing the welding speed.

**Figure 5:**
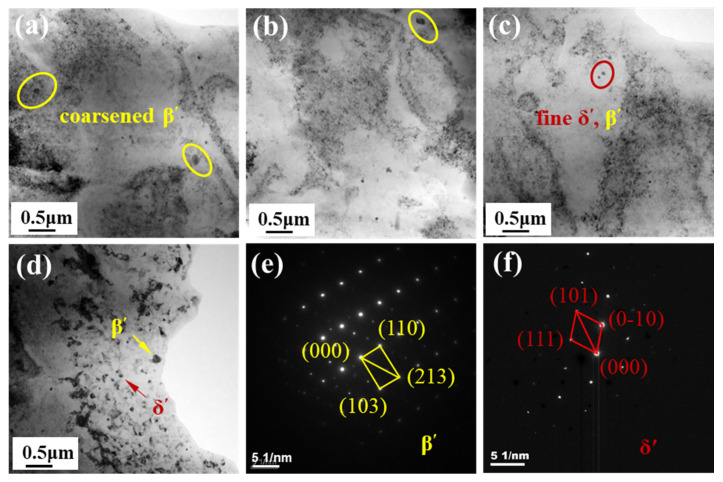
Distribution of reprecipitated phase particles in the NZ: (**a**) 90 mm/min; (**b**) 120 mm/min; (**c**) 150 mm/min; (**d**) 180 mm/min; (**e**) SAED of β’ phase; (**f**) SAED of δ’ phase.

Figure 5 shows reprecipitated phase particles distribution in the weld NZ under different welding speeds. Clearly, the strengthening phase particles vary greatly. Among them, at a higher welding speed of 180 mm/min, a large number of the particles appear in the NZ in Figure 5d. With the welding speed decreasing to 150 mm/min, the phase particle distribution obviously changes and the size of the particles is finer. By the calibration of the diffraction pattern in Figure 5e,f, it is found that these fine particles are mainly composed of the reprecipitated δ’ and β’ phases. Under the condition, the welding speed gradually decreases to 120 mm/min and 90 mm/min, and some fine particles in the NZ begin to grow up and form coarse β’ phase, as shown in Figure 5a,b.

Similarly, the reprecipitated behavior of the strengthening phase particles is related to the welding temperature during FSW. At 180 mm/min, the welding temperature is too low and can lead to insufficient plastic degree of the weld metal. Original δ’ and β’ phase particles in Al–Li alloy are not fully broken, dissolved [25] and reprecipitated [26], and coarse strengthening phase particles are found in the NZ. By increasing the welding temperature, the weld metal is sufficiently plasticized due to a lower welding speed of 150 mm/min, and many fine δ’ and β’ phases particles are reprecipitated. However, when the welding temperature is too high, some fine reprecipitated particles in the NZ may start to grow abnormally.

### 3.3. Microhardness

**Figure 6:**
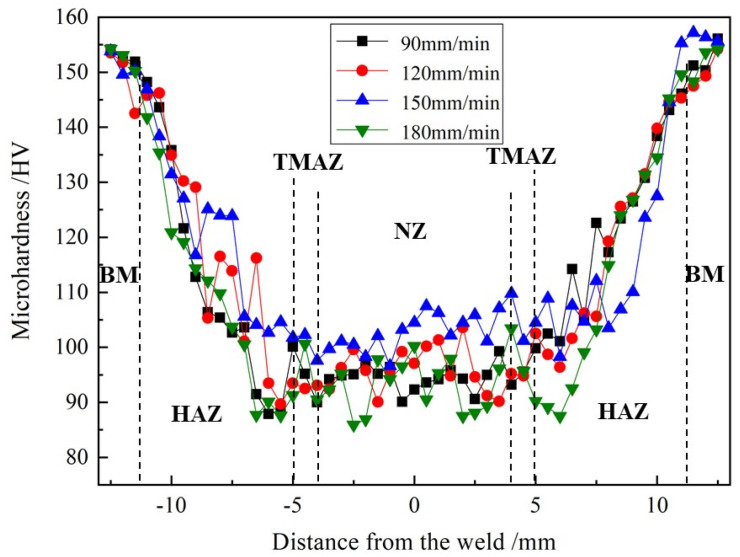
Microhardness of different FSW joints.

Figure 6 shows the microhardness of all FSW joints fabricated by different welding speeds. It is observed that the microhardness distribution of the joints presents a “W” shape, which indicates that the microhardness in the TMAZ and HAZ gradually decreases. The average value is minimal in the HAZ. In addition, compared with the NZ, in TMAZ and HAZ, the microhardness value of the base material is the highest, demonstrating that three zones in the weld are obviously softened. It is also found that the maximum average microhardness of the NZ is about 103 HV, made by a 150 mm/min welding speed. However, the average microhardness is only 93 HV at 180 mm/min, which is the lowest.

In general, the microhardness distribution of FSW joints is related to their microstructure [27]. The main reason is that the original particles of strengthening phases in the Al–Li alloy are basically dissolved in the FSW process [28], and only part of them are reprecipitated in the NZ after welding. So, the microhardness value of the NZ is much lower than that of the base metal. On the other hand, it is higher than that of the TMAZ and HAZ, attributed to dynamic recrystallization caused by the stirring action of rotating tool, forming finer equiaxed grains and reprecipitated particles. In addition, according to the Hall–Petch principle [29] and the Orowan strengthening mechanism [30], when the welding speed used is 150mm/min, the average value of the microhardness in the NZ is the highest due to the finest equiaxed grains and reprecipitated particles. On the contrary, the average value is the lowest, resulting from a coarse microstructure at 180 mm/min in Figure 3 and Figure 5.

### 3.4. Mechanical Properties

#### 3.4.1. Tensile Properties

**Figure 7:**
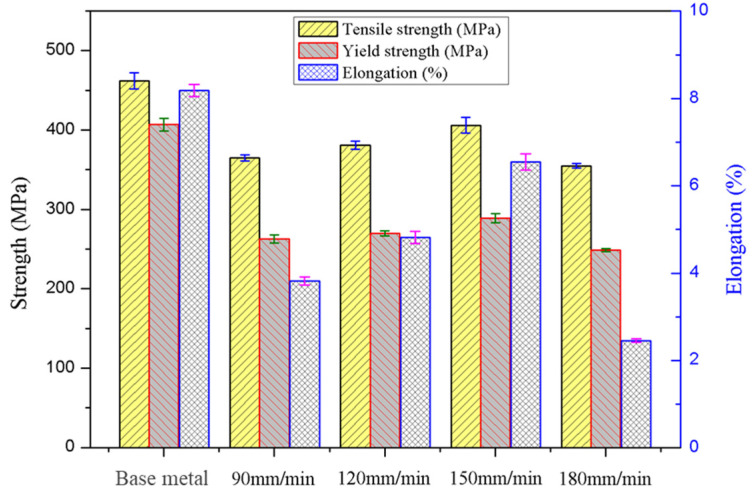
Tensile properties of FSW joints produced at various welding speeds and base material.

Figure 7 presents the tensile testing results of different FSW joints and base metal. It is clearly seen that the tensile properties of all FSWed joints were significantly reduced compared with the base metal. Moreover, with the welding speed decreasing from 180 mm/min to 90 mm/min, the tensile strength presents first an increasing and then decreasing trend. The mechanical performances, such as the tensile strength (TS), yield strength (YS) and elongation (EL) of FSW joints, obtained at 150 mm/min are the best, which are 406 MPa, 289 MPa and 7.2%, respectively. Whereas, the minimum value of TS, YS and EL of the joints is, respectively, 355 MPa, 249 MPa and 2.7%, when a 180 mm/min welding speed is used during FSW. This is consistent with the microstructure and microhardness of the different joints mentioned above.

For a precipitation strengthening Al alloy, the mechanical properties have a close relation to the grain size and precipitated phase particles [31]. In the base material, there is the presence of a large quantity of precipitated phase, and the strength and elongation of the base material present a higher value. For the NZ, the fine equiaxed grains can contribute to a high property. However, most of the precipitated phase particles are dissolved in the NZ during FSW, as observed in Figure 5. The active contribution from the fine grains to tensile properties cannot recover the obviously negative impact of the dissolution of precipitated phase particles. In consequence, the tensile properties of all FSW joints are lower than those of the base material. On the other hand, there exist significant differences in the grain size and reprecipitated phase distribution of different FSW joints produced by different welding speeds. Using a 150 mm/min welding speed, the positive contribution from finer equiaxed grains and more reprecipitated particles into the NZ can improve the mechanical properties of FSW joints. However, it is an opposite result derived from the evidently negative effect of coarse grain and reprecipitated phase particles in the NZ for the joints fabricated at 180 mm/min.

#### 3.4.2. Fractured Locations

**Figure 8:**
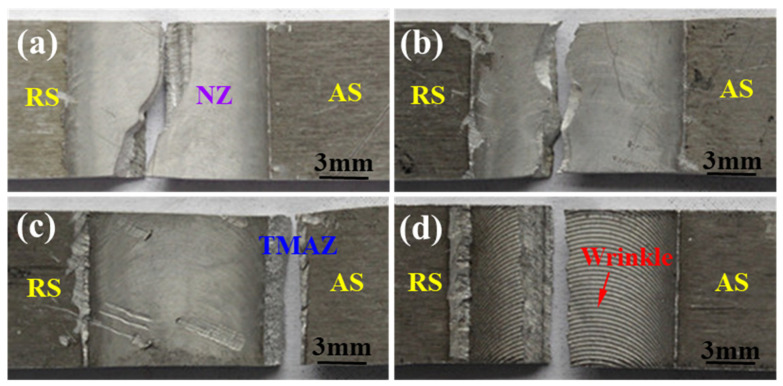
Fractured locations of tensile test joints: (**a**) 90 mm/min; (**b**) 120 mm/min; (**c**) 150 mm/min; (**d**) 180 mm/min.

Figure 8 shows the fractured positions of all joints obtained at various welding speeds after tensile testing. It is clear that that the joint produced at 150 mm/min fractures in the TMAZ with an obvious necking phenomenon, as seen in Figure 8c. However, other joints are all fractured in the NZ (in Figure 8a,b,d), when the welding speeds of 180 mm/min, 120 mm/min and 90 mm/min are used during the FSW process.

#### 3.4.3. Fractured Surface

**Figure 9:**
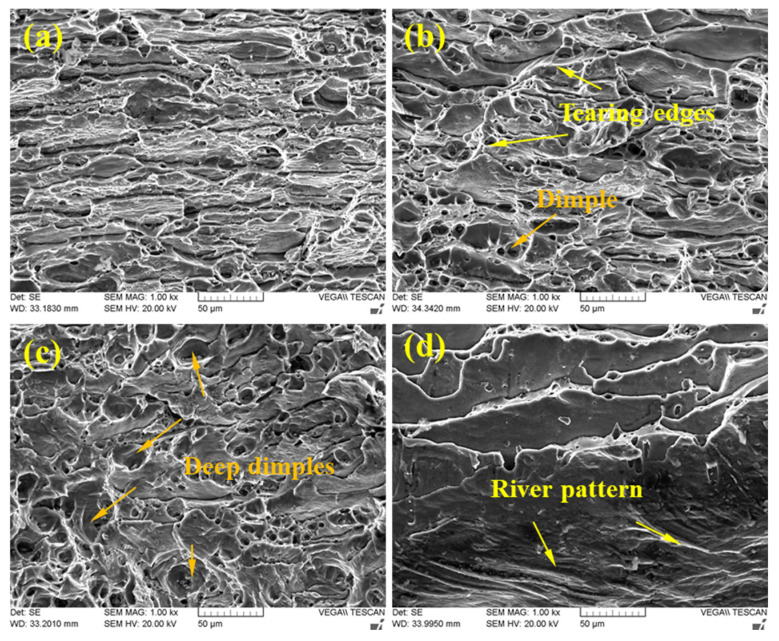
SEM images of fracture surface for different tensile test joints: (**a**) 90 mm/min; (**b**) 120 mm/min; (**c**) 150 mm/min; (**d**) 180 mm/min.

Figure 9 shows SEM images of the fractured surface of all tensile joints produced by different welding speeds. As seen in Figure 9d, a large number of river patterns and cleavage platforms are found on the fractured surface of the joint obtained at 180 mm/min, suggesting that it is a typical brittle fracture. With the welding speed decreasing to 150 mm/min, there exists a large number of deep dimples and tearing ridges on the fractured surface (Figure 9c), indicating that this fracture mode is a complete ductile fracture. For FSW joints of 120 mm/min and 90 mm/min, fine dimples and few tearing ridges on the flat facets indicate a mixed fracture mode appearing in the NZ, as shown in Figure 9a,b.

## 4. Conclusions

The 2198 Al-Cu-Li alloys have been successfully joined by friction stir welding at different welding speeds with an invariable rotation speed. The microstructure evolution and mechanical properties of the joints are investigated. The main conclusions are as follows:

1. With the welding speed decreasing from 180 mm/min to 90 mm/min, the size of the nugget zone (NZ) first increases and then decreases due to different welding temperatures. At 150 mm/min, the size is the biggest and the “S” curve in the NZ starts to disappear, resulting from sufficient plastic flow;
2. The NZ is composed of fine equiaxed grains for all joints. Among them, the equiaxed grains are finer, attributed to a higher degree of dynamic recrystallization, when a welding speed of 150 mm/min is used. A larger number of fine reprecipitated phase (δ’, β’ phases) particles are dispersively distributed in the NZ, which can result in higher microhardness and better tensile properties of the joins;
3. Compared to the base material, the average microhardness of the NZ is reduced as a greater amount of δ’ and β’ phases particles is dissolved into the NZ, which makes a more obviously negative impact recover an active contribution from finer grains;
4. The joints obtained at 150 mm/min have the highest tensile properties, and the tensile strength, yield strength and elongation are, respectively, 406 MPa, 289 MPa and 7.2%, which is consistent with the microstructure and microhardness of the joints. Only the joints at 150 mm/min are fractured in the TMAZ, with significant necking phenomenon. Moreover, deep dimples and many tearing ridges are detected on the fracture surface, indicating a complete ductile fracture mode.

## Figures and Tables

**Table 1:** Chemical compositions of base material.

Alloy	Mass Fracture (%)					
	Cu	Li	Mg	Mn	Zr	Al
2198-T8	2.9–3.5	0.8–1.1	0.25–0.8	0.1–0.5	0.04–0.18	Bal

## Data Availability

Data are contained within the article.
